# Epigenetic modulation of immunotherapy and implications in head and neck cancer

**DOI:** 10.1007/s10555-020-09944-0

**Published:** 2021-01-05

**Authors:** Liye Zhou, Na Xu, Hirofumi Shibata, Vassiliki Saloura, Ravindra Uppaluri

**Affiliations:** 1 Department of Medical Oncology, Dana-Farber Cancer Institute, Boston, MA USA; 2 Department of Tea and Food Science, Anhui Agricultural University, Hefei, Anhui People’s Republic of China; 3 Department of Otolaryngology, Gifu University Graduate School of Medicine, Gifu, Japan; 4 Center for Cancer Research, National Cancer Institute, Bethesda, MD USA; 5 Department of Surgery/Otolaryngology, Brigham and Women’s Hospital, Boston, MA USA

**Keywords:** Head and neck cancer, Epigenetics, Tumor microenvironment, Immunotherapy

## Abstract

Cancer progression is facilitated by distinct mechanisms developed by cancer cells to avoid immune recognition and clearance. The clinical application of immune checkpoint blockade (ICB), *via* monoclonal antibodies blocking PD-1/PD-L1 and CTLA4, has achieved promising durable therapeutic response in various cancer types, including recurrent and metastatic head and neck squamous cell carcinomas (HNSCC). HNSCC represents a rational target of ICB treatment given its relatively high mutation burden and the presence of immune infiltrates. However, the limited response rates and recent negative clinical trials data identify an urgent need for new strategies to overcome immunotherapy resistance. Preclinical studies have revealed an important contribution of epigenetic regulators in the anti-tumor immune response. Multiple components of the tumor and host immune system interaction are under epigenetic regulation, including the cancer cells themselves, cytotoxic T lymphocytes, regulatory T lymphocytes, natural killer cells, and tumor-associated macrophages. Epigenetic targeting drugs such as DNA methyltransferase inhibitors, histone deacetylase, and methyltransferase inhibitors have demonstrated the potential to reverse immune suppression in various cancer models. The aim of this review is to summarize recent preclinical studies focused on investigating the function of epigenetic modulation in the host immune and cancer cell interface. We also provide a perspective on combining epigenetic modulation and immunotherapy in the management of HNSCC to improve outcomes—an area of great interest in future clinical studies.

## Introduction

Immunotherapy has emerged as one of the most exciting clinical frontiers in cancer management over the latest decade and has given new hope to poor prognosis cancer patients. Immune checkpoint blockade, including inhibition of the programmed death 1 (PD-1) receptor pathway or CTLA4/CD152, aims to reinvigorate the host anti-tumor immune response. Blocking these pathways results in an effector T cell response leading to cancer cell eradication [1–3]. Despite impressive results from multiple clinical trials demonstrating a durable response, only a fraction of patients in most cancer types are responsive to immune checkpoint blockade treatment [4–9].

Head and neck squamous cell carcinomas (HNSCC) represent one of the tumor types where up to 80% of patients do not respond to anti-PD-1 based therapies. HNSCCs are the sixth most common cancer worldwide and can be divided into a classical carcinogen (tobacco and alcohol) induced variety and one where the human papillomavirus (HPV) is the primary etiology. Both HPV-positive and HPV-negative HNSCC tumors are highly immune infiltrated [10]. The HNSCC mutation burden is at the relatively higher end within the spectrum across human cancer types, which usually predicts a higher number of mutation derived neoantigens, the ultimate target of the immune system [11, 12]. The use of PD-1 blockade with nivolumab or pembrolizumab in second-line recurrent and metastatic HNSCC was approved by the US Food and Drug Administration (FDA) in 2016 [13]. Recent data from KEYNOTE-048 have expanded the use of pembrolizumab to first-line recurrent and/or metastatic HNSCC. However, the response rates of only 15–20% highlight the need for continued investigation to advance therapeutic options [14–16].

Circumventing resistance mechanisms to immunotherapy represent a robust research area that is the critical barrier to improve the clinical outcomes of cancer patients [17, 18]. Mechanisms of resistance can be divided into (a) tumor cell intrinsic immunoevasive genetic pathways, (b) a suppressive tumor microenvironment, or (c) host factors including somatic variants and the gut microbiome [19]. A major cancer cell resistance mechanism involves lesions in genes in the interferon-gamma (IFNγ) signaling pathway or antigen presentation machinery that independently impair the efficacy of immunotherapy [20–23]. High infiltration of immunosuppressive regulatory T cells (Tregs) and myeloid-derived suppressor cells (MDSCs) in the tumor microenvironment is associated with a poor prognosis in various cancers [24, 25]. Extending the work in mouse models [26], a correlation between the gut microbiome and efficacy of immune checkpoint therapy has been shown in melanoma, lung, and kidney cancer patients [27–29].

Various combinatorial therapies have been proposed to improve the response rate and overcome resistance to anti-PD-1 treatment. These strategies include combining with other immunotherapies, chemotherapy, targeted therapeutics, or radiation [30–40]. Several recent negative trials have unfortunately given pause on next steps in combination approaches. The EAGLE Phase III trial that evaluated combining durvalumab with tremelimumab *versus* standard of care chemotherapy failed to show an enhanced clinical impact in recurrent/metastatic HNSCC [41]. The Javelin Head and Neck 100 Phase III trial combining avelumab with chemoradiotherapy in the definitive HNSCC setting was closed early as there was no enhanced activity relative to chemoradiation [42]. These negative results of Phase III trials have highlighted that exploration of rational combinations beyond existing therapies is needed.

The ideal therapy to combine with anti-PD-1 agents would be one that has pleiotropic effects on multiple targets that together would limit development of resistance. As such, therapeutics that target epigenetic modifications of both cancer cells and components in the tumor immune microenvironment represent such an opportunity. In this review, we focus on recent preclinical studies with epigenetic modulation in cancer immunotherapy and discuss the effects of epigenetic interventions in cancer cells, T-cells, natural killer cells, and macrophages. We review the potential of existing epigenetic therapeutics in promoting antitumor immunity followed by a brief discussion of strategies to define new therapeutic targets. Finally, we describe the rationale for combining epigenetic targeting and immunotherapy to improve the clinical outcomes of HNSCC.

## Epigenetic therapeutics

In broad terms, epigenetic alterations refer to gene expression changes resulting from effects of DNA methylation, histone modification, regulatory non-coding RNA, or transcription factors and not caused by alteration in DNA sequences. Cancer cells are highly enriched for epigenetic abnormalities, which makes epigenetic regulators attractive targets in cancer treatment. DNA methylation, histone acetylation, and histone methylation, specifically EZH2-mediated histone H3 lysine 27 tri-methylation (H3K27me3), represent the major targets where therapeutics is available for epigenetic therapy.

The inhibition of DNA methyltransferases (DNMTs) targets promoter region hypermethylation on the cytosine residues. DNMT inhibitors (DNMTis), such as 5-azacytidine and 5-aza-2′-deoxycytidine, have been approved by the United States Food and Drug Administration (FDA) in the treatment of patients with myelodysplastic syndrome (MDS) and acute myeloid leukemia (AML) [43–45].

Post-translational histone modifications include histone acetylation and methylation. Histone acetylation increases the accessibility of chromatin to the transcriptional machinery by removing the positive charge on the histones. Blocking of HDAC activity using small molecule inhibitors has shown potent effects in suppressing tumor progression and apoptosis of cancer cells. HDAC inhibitors, such as Vorinostat, Belinostat, and Panobinostat have been approved for the treatment of cutaneous T cell lymphoma, chronic lymphocytic leukemia, and multiple myeloma, respectively [45, 46]. Vorinostat, also known as suberanilohydroxamic acid (SAHA), is one of the most advanced small molecule pan-HDAC inhibitors, which is administered orally [47].

Enhancer of Zeste 2 Polycomb Repressive Complex 2 Subunit (EZH2) is the catalytic component in the Polycomb Repressive Complex 2 (PRC2), which tri-methylates lysine 27 of histone H3 (H3K27me3). The inhibition of EZH2 activity may slow tumor growth by upregulating tumor suppressor gene expression. The clinical efficacy of an orally administered EZH2 inhibitor, tazemetostat, is under investigation in multiple clinical trials including non-Hodgkin lymphoma (NHL), INI1/SMARCB1-negative tumors, synovial sarcoma, colorectal cancer, prostate cancer, renal cell carcinoma, ovarian cancer, and mesothelioma. Tazemetostat has recently been approved for the treatment of metastatic or locally advanced epithelioid sarcoma and relapsed or refractory (R/R) follicular lymphoma (FL) whose tumors are positive for an EZH2 mutation.

## Cancer cell epigenetic modulation

The goal of cancer immunotherapy is to modify the interface between cancer cells and immune compartments in the microenvironment to support an anti-tumor response. We first discuss recent preclinical studies on epigenetic modulation in cancer cells that may facilitate the antitumor response by impacting innate and adaptive immune pathways.

### Modulation of innate immune responses

Epigenetic regulation of innate immune responses where Type I interferons are induced can lead to enhanced anti-tumor immunity and promote the efficacy of anti-PD-1 treatment. The lysine-specific histone demethylase 1A (LSD1), a histone H3K4 demethylase, is a negative regulator of endogenous retroviral element (ERV) expression, and its function in anti-tumor immunity was defined in a compound screen using MCF7 cells [48]. Inhibition of LSD1 caused the accumulation of intracellular double-stranded RNA (dsRNA), which was sensed by Toll-like receptor 3 (TLR3) and melanoma differentiation-associated protein 5 (MDA5) that subsequently triggered type I IFN activation. LSD1 deficiency in cancer cells triggered antitumor T cell responses and overcame anti-PD-1 resistance in the murine B16 melanoma model [48].

DNMTi, including 5-Azacytidine or 5-aza-2-deoxycytidine, targeted colorectal cancer-initiating-cells and ovarian cancer by activating an interferon response *via* double-stranded RNA triggered viral defense and downstream MDA5/MAVS/IRF7 activation [49, 50]. Moreover, the viral defense gene signature in tumor samples significantly correlated with the long-term clinical outcome of melanoma patients treated with anti-CTLA4 [50], and combination of DNMTi with anti-CTLA4 in the murine B16 melanoma model showed favorable therapeutic response [50]. Inhibition of DNMT using 5-azacytidine resulted in increased interferon signaling, cancer testis antigen genes, antigen processing and presentation machinery, cytokines and chemokines in colon, breast, and ovarian cancer cell lines [51, 52]. In addition, DNMTi *in vitro* treatment enhanced the expression levels of viral defense genes and endogenous retroviral transcripts in both human and mouse epithelial ovarian cancer lines [53]. The *in vivo* anti-tumor effect of 5-azacytidine was dependent on an intact Type I IFN signaling pathway in epithelial ovarian cancer. Furthermore, a triple combination of DNMTi, HDACi, and anti-PD1 showed an optimal therapeutic effect [53]. In non-small-cell lung cancer (NSCLC), Baylin and colleagues showed that *in vitro* combinatorial treatment of HDACi and DNMTi resulted in the suppression of MYC signaling, as well as the induction of Type I IFN pathway related genes and antigen presentation genes [54]. The combinatorial epigenetic treatment in NSCLC murine model reduced tumor burden and increased CD8+ T cell infiltration in the tumor microenvironment [54].

### Modulation of adaptive immune responses

**Fig. 1:**
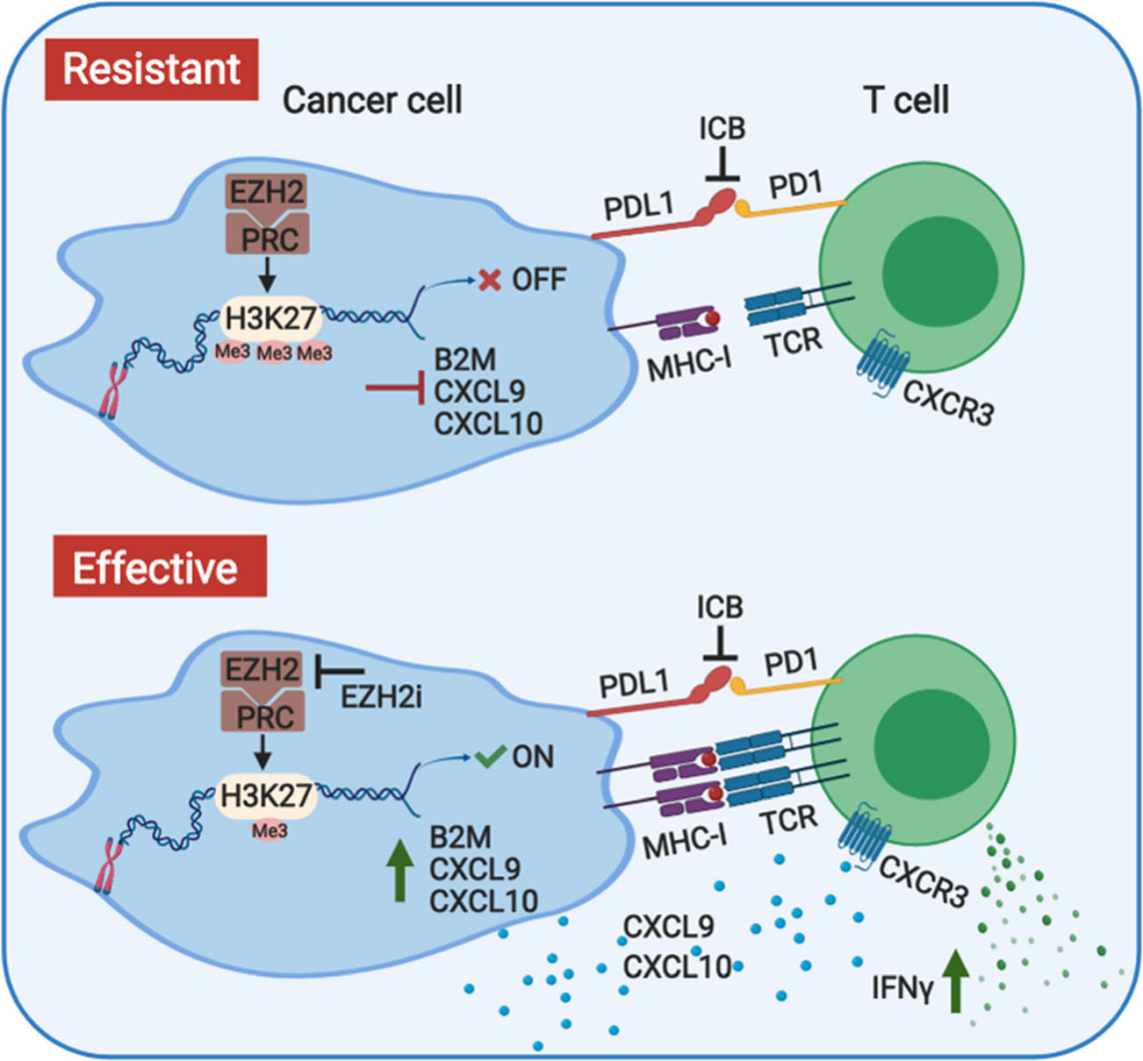
Ezh2 inhibition promotes the responsiveness of anti-PD1 therapy in HNSCC. EZH2 regulated H3K27me3 modification on the promoter of β-2-microglobulin to modulate its gene expression levels. Targeting of Ezh2 enhances both antigen presentation machinery and T cell recruiting chemokines, CXCL9 and CXCL10, production in HNSCC models. Combination of EZH2 inhibition and immune checkpoint blockade significantly promotes cancer cell/T cell recognition as well as T cell recruitment in the tumor. Therefore, targeting Ezh2 in combination with anti-PD1 therapy might enhance the efficacy of immunotherapy in patients with HNSCC

Enhanced adaptive immune responses *via* epigenetic targeting can occur through enhanced T cell recognition and recruitment by cancer cells. We and others have identified that EZH2 targeting can enhance adaptive immune responses. As impaired antigen presentation is common in HNSCC [55], we reasoned that enhancing antigen presentation by cancer cells would be a rational strategy to promote responsiveness to anti-PD-1 therapy in HNSCC. We first observed a negative correlation between the expression levels of EZH2 and major class I antigen presentation molecules in the TCGA HNSCC cohort. Therefore, we hypothesized a regulatory function of EZH2 in HNSCC antigen presentation [56]. Both genetically attenuated EZH2 expression and pharmacologic EZH2 inhibition resulted in significantly higher class I expression and antigen presentation capacity (Fig. 1). Functionally and as expected, this resulted in elevated antigen-specific T cell proliferation and cytokine production. Mechanistically, we found that EZH2 regulated H3K27me3 modification on the promoter of β-2-microglobulin to modulate gene expression (Fig. 1). In addition, in two murine HNSCC transplantable cell line models, we confirmed *in vivo* upregulation of tumor cell antigen presentation induced by EZH2 inhibition. More importantly, the combinatorial treatment of an EZH2 inhibitor and anti-PD1 significantly suppressed tumor progression of an anti-PD-1 resistant HNSCC model [56]. Thus, our preclinical study highlighted the potential of combined EZH2 inhibition and anti-PD-1 treatment in improving the efficacy and clinical outcome of immunotherapy in patients with HNSCC (Fig. 1).

Several additional studies also identified that EZH2 inhibition augments class I expression. In B16 or RIM-3 melanoma cells, EZH2 inhibition resulted in upregulation of antigen presentation molecule expression *via* modulating H3K27me3 modification on promoter regions [57]. Dawson and colleagues showed that EZH2 represents a conserved mechanism in class I presentation when they identified the regulatory role of PRC on class I antigen presentation from a genome-wide CRISPR screen [58]. EZH2 inhibition induced upregulation of class I antigen presentation in multiple MHC-I low cancer types, which subsequently sensitized cancer cells to T cell-mediated killing. This elegant work has demonstrated the conserved function of PRC in negatively regulating the antigen presentation process in cancer cells and the potential of EZH2 inhibition in augmenting antitumor immunity (Fig. 1) [58]. In addition, inhibition of DNMT has been shown to be able to upregulate breast cancer cell class I antigen presentation levels and promote the efficacy of anti-PD-L1 therapy in murine models [59].

By contrast, in ovarian cancer models, Zou and colleagues found that EZH2 inhibition did not alter class I antigen presentation of ovarian cancer cells, which indicates that the regulation of EZH2 on antigen presentation may be cancer-type specific [60]. Instead, this study showed that both EZH2-mediated H3K27me3 modification and DNMT1-mediated DNA methylation suppressed the expression of CXCL9 and CXCL10 in cancer cells [60]. CXCL9 and CXCL10 are the major Th1 chemokines responsible for T cell recruitment to the tumor microenvironment [28]. EZH2 and DNMT inhibitor treatment increased T cell infiltration in murine models of ovarian cancer and improved the efficacy of immunotherapies. We observed a consistent dramatic induction of CXCL9 and CXCL10 expression by the EZH2 inhibition in human but not murine HNSCC cell lines (Fig. 1) [56]. Together, these data highlight the potential dual function of Ezh2 inhibition on promoting cancer cell antigen presentation and Th1 chemokine production in human HNSCC (Fig. 1).

## T cell epigenetic modulation

A significant subset of immunotherapies, including immune checkpoint blockade, seek to directly modulate T cell function to eradicate cancer cells. An effective anti-tumor T cell response requires TCR signaling pathway stimulation, clonal expansion, and differentiation to effector functionality, in which epigenetic modulation plays a key role. Therefore, the idea of therapeutically targeting T cell epigenetic programs is of great interest in achieving better clinical outcomes.

### Modulation of CD8+ T cell function

Studies on the steps in T cell differentiation in viral infection models have highlighted the critical contribution of epigenetic remodeling *via* PRC2-mediated H3K27me3 marks in maintaining memory-like characteristics [61, 62]. Antigen-specific terminally differentiated effector T cells (KLRG1^hi^IL-7R^lo^) showed high levels of H3K27me3 deposition preferentially at pro-memory genes to restrict memory fates compared to memory precursor T cells (KLRG1^low^IL-7R^hi^). The expression of EZH2 in T cells is also induced by TCR signaling upon activation. EZH2 is required for CD8+ T cell clonal expansion and terminal effector differentiation in a productive anti-viral response [61]. Also, impaired EZH2 expression in T cells resulted in poor tumor control in ovarian cancer and melanoma, highlighting the important role of Ezh2 in maintaining the survival of effector T cells and T cell polyfunctional cytokine expression as well as the formation of memory precursor T cells [63, 64]. Akt-mediated phosphorylation of Ezh2 in T cells resulted in reduced formation of memory precursor T cells, suggesting Akt-mediated phosphorylation of Ezh2 as a target to potentially enhance anti-tumor T cell response [64]. Hence, the crucial role of Ezh2 in maintaining the functionality of effector T cells and formation of memory precursor T cells emphasizes the potential detrimental effects of targeting Ezh2 in T cells on the outcome of cancer immunotherapy.

**Fig. 2:**
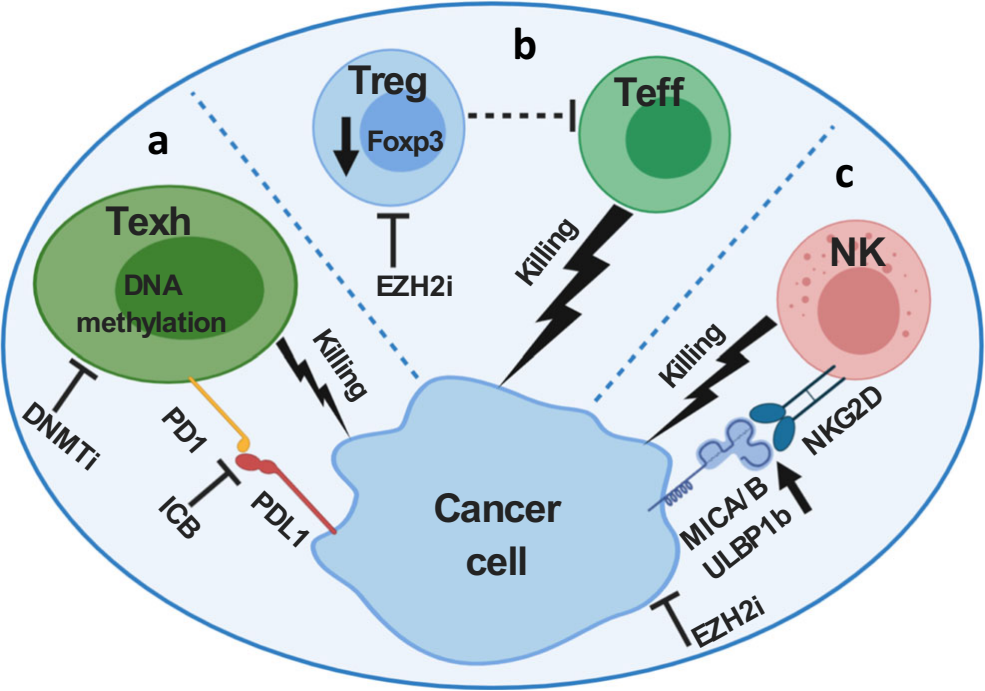
Epigenetic modulation involved in the functionality of various components in the tumor immune microenvironment. **a** Sequential DNMTi and anti-PD1 treatment reverses the T cell exhaustion associated *de novo* DNA methylation program, which promotes antigen specific T cell expansion and better control of tumor growth. **b** Treg-specific inhibition of Ezh2 decreased Foxp3 protein stability, hence Ezh2 deficiency in Tregs promoted antitumor immunity with enhanced cytotoxic T cell infiltration and effector function. **c** Inhibition of cancer cell intrinsic Ezh2 inhibition augmented cancer cell/NK cell recognition by increasing the expression of NKG2D ligands, including ULBP1-6, MICA, and MICB. Ezh2 inhibitor treatment increased NK cell infiltration as well as NK cell-mediated killing of cancer cells

The concept of T cell exhaustion was first introduced in antigen-specific T cells in chronic infection with the lymphocytic choriomeningitis virus (LCMV) model. This model shows significant parallels with tumor-associated-antigen specific T cell responses [65]. T cell exhaustion, as a result of chronic antigen exposure, is a developmental stage with a unique epigenetic profile distinct from effector or memory T cells [66, 67]. PD-1/PD-L1 blockade has been proposed to act by disrupting the interaction between PD-1 and PD-L1 to reinvigorate dysfunctional exhausted T cells. However, the reprogramming of exhausted T cells by anti-PD-L1 is limited to transient transcriptomic changes without changing the epigenetic landscape, which could limit the efficacy of immune checkpoint blockade in cancer patients [66]. A *de novo* DNA methylation program was found to be induced by chronic TCR signaling in exhausted T cells, and this DNA methylation program was also not reversible by PD-1 blockade (Fig. 2) [68]. The administration of DNA demethylating agent, 5-aza-2′-deoxycytidine before the treatment with PD-1 blockade significantly reversed the exhaustion associated *de novo* DNA methylation program. Sequential 5-aza-2′-deoxycytidine and anti-PD-1 treatment synergistically promoted antigen-specific T cell expansion in both viral and tumor models, which resulted in better control of tumor growth (Fig. 2) [68].

### Modulation of Treg function

EZH2 also has a critical role in maintaining the identity and function of regulatory T cells (Tregs) in preventing autoimmunity [69, 70]. In the context of tumor immunology, the function of EZH2 in Tregs was investigated by Dupage and Sharma, respectively (Fig. 2) [71, 72]. Both studies used mouse models that selectively deleted *Ezh2* in Tregs finding that *Ezh2* deficiency in Tregs promoted antitumor immunity with enhanced T cell infiltration and elevated effector function [71, 72]. Mechanistically, Ezh2 functioned in regulating Foxp3 protein stability (Fig. 2) [71]. Moreover, temporal genetic deletion of *Ezh2* in Tregs showed better control of implanted murine prostate TRAMPC2 and colon adenocarcinoma MC38 tumor growth without causing auto-immune toxicities. Surprisingly, the potency of temporal Treg *Ezh2*-deficiency was higher than systemic depletion of Tregs in controlling tumor progression, indicating a potential pro-inflammatory function of Ezh2-deficient Tregs [71].

Pharmacologic Ezh2 inhibition *via* CPI-1205 treatment repressed *in vitro* differentiation and suppressive capacity of both human and mouse Tregs [72]. In addition, treatment of CPI-1205 induced a proinflammatory transcriptomic signature, as well as higher proinflammatory cytokine production by Tregs. Ipilimumab treatment in patients with metastatic prostate cancer increased EZH2 protein expression levels in peripheral CD4+ T cells compared with pre-treatment basal levels. Therapeutically, *in vivo* studies using mice bearing the murine bladder cancer cell line MB49 showed that systemic administration of CPI-1205 in combination with anti-CTLA4 resulted in better anti-tumor immunity compared with anti-CTLA4 single therapy [72].

As reviewed above, Tregs-specific targeting of Ezh2 resulted in impairment of immunosuppressive function, hence promoting CD8+ T cell antitumor activity. This finding has to be balanced with the direct impact of Ezh2 inhibition and compromised CD8+ T cell effector function. Therefore, the overall effect of Ezh2 inhibition on cancer cell/T cell interaction is the result from summation of Ezh2 inhibition on Treg and CD8+ T cell function. Using the same ovarian cancer model, Zou and colleagues showed that Ezh2 deficiency in T cells suppressed control of tumor progression [63], while combined Ezh2 and DNMT inhibitors improved the efficacy of anti-PD-1 therapy by increasing the production of CXCL9 and CXCL10 [60]. A possible explanation is that the attenuation of the CD8 T cell function by Ezh2 inhibition is overcome by DNMT inhibitor induced immunostimulatory factors, such as CXCL9 and 10, in the tumor microenvironment. In our HNSCC anti-PD-1 resistant model, systemic administration of Ezh2 inhibitor in combination with anti-PD-1 resulted in suppressed tumor progression [56]. Our finding of tumor cell enhanced antigen presentation machinery and antigen-specific CD8+ T cell cytotoxicity potentially may have overcome the dampening effect of Ezh2 inhibition on the function of CD8+ T cells. It is also possible that the impairment of CD8 T cell function by Ezh2 targeting varies among different cancer types. Therefore, further cancer type-specific preclinical studies are crucial in defining the complex functions of epigenetic modulators.

### NK cell epigenetic modulation

The development and maturation of NK cells are subject to histone modification regulation [73]. In *Ezh2* deficient mice, the frequency of NK cells was increased in multiple organs, including the spleen, liver, and bone marrow [73]. Selective inhibition of Ezh2 *in vitro* resulted in enhanced NK cell development and increased expression of a major activating receptor, NKG2D, on NK cells [73]. A small molecule inhibitor screen for epigenetic regulators of pro-inflammatory cytokine production in NK cells highlighted the critical role of H3K27me3 in NK cell activation [74].

The epigenetic modulation of NK cells in cancer immunology has been extensively reviewed elsewhere [75]. Here we highlight the importance of histone H3K27me3 modification in the NK cell-mediated eradication of cancer cells. Treatment with the EZH2 inhibitor,EPZ011989, in muscle-invasive bladder cancer bearing nude mice increased NK cell infiltration and activation in the tumor specimens [76]. Wajapeyee and colleagues sought to identify epigenetic modulators of NKG2D ligand expression in hepatocellular cell carcinoma (HCC) that may facilitate NK cell-mediated killing [77]. NK cells respond to target cells *via* a balance of interactions between activating and inhibitory receptors and their ligands [78]. Selective inhibition of EZH2 in multiple HCC cell lines significantly increased the expression of NKG2D ligands, including UL16-binding protein 1–6 (ULBP1–6), major histocompatibility complex class I chain-related gene A (MICA), major histocompatibility complex class I chain-related gene B (MICB), CD112, and CD155 (Fig. 2). In addition, genetic ablation and pharmacological inhibition of EZH2 enhanced NK cell-mediated killing of HCC cells. Mechanistic studies revealed that EZH2 enhanced the methylation of ULBP1-6 promoter region by recruiting DNMT3A, which subsequently suppressed the expression of ULBP1-6 (Fig. 2) [77]. Collectively, these preclinical studies suggested that targeting of EZH2 in both NK cells and cancer cells can potentially promote the NK cell-mediated killing of cancer cells.

## TAM repolarization using epigenetic modulation

Tumor associated macrophages (TAMs) are distributed in a polarization spectrum from classically activated M1- macrophages (M1s) to alternatively activated M2-like macrophages (M2s). M1s and M2s have opposite functions in tumor progression: M1s are pro-inflammatory tumor-inhibiting, while M2s are immunosuppressive tumor-supporting [79]. High infiltration of M2s correlates with poor prognosis of HNSCC [80, 81]. Therefore, repolarization of TAMs towards M1s represents a major strategy in manipulating TAM to control tumor progression. Interferon gamma (IFNγ), lipopolysaccharide (LPS), and other Toll-like receptor ligands favor M1 polarization. Ivashkiv and colleagues demonstrated that IFNγ induced EZH2-mediated histone H3K27me3 modifications at the promoter regions of anti-inflammatory genes suppressed the gene expression in primary human monocytes-derived macrophages. In addition, IFN γ-induced H3K27me3 resulted in the stable suppression of gene expression [82]. Therefore, EZH2 is involved in the chromatin remodeling process induced by IFNγ to repress anti-inflammatory gene expression in macrophages to achieve and maintain the activated M1 state [82].

SOCS1 (suppressor of cytokine signaling 1), a key cytokine signal negative regulator has been shown to be under epigenetic modulation in macrophages [83]. Cheng and colleagues found that DNMT1 mediated DNA methylation in the promoter region of SOCS1 resulted in the suppression of its gene expression. Inhibition of DNMT activity using 5-aza-2′-deoxycytidine in LPS-activated RAW264 macrophage cells reduced SOCS1 expression and consequently enhanced the production and release of pro-inflammatory cytokines such as TNFα and IL-6 [83].

## New immune frontiers in epigenetics

Further granular details about the interaction between tumor and host immune compartments are being revealed in expanded studies of cancer immunotherapy response including by the use of innovative technology platforms. Together, these approaches will provide invaluable information in developing new therapeutic targeting strategies.

For example, the anti-tumor contribution of tumor infiltrating B cells has been controversial [84–87]. Recently, the association between the presence of B cells in tertiary lymphoid structures in tumors and favorable responses to immunotherapy has been demonstrated in soft tissue sarcoma, metastatic melanoma, and renal cell carcinoma, respectively [88–90]. Thus, different B cell subsets in the tumor microenvironment may have distinct contributions in an antitumor immune response [91]. The effect of epigenetic modulation in B cells to cancer immunotherapy response remains to be explored. However, the differentiation and activation of B cells are under the regulation of histone and DNA modification, such as H3K27me3, H3K9me3, and DNA methylation [92]. It will be of interest to investigate the effect of epigenetic manipulation on the spatial distribution and functional contributions of B cell subsets in tumors.

## Novel target identification strategies in cancer immuno-epigenetics

Development of CRISPR/Cas9 systems for ablation of specific gene function in large-scale discovery screening has allowed unbiased interrogation of critical immune pathways. Using genome-wide CRISPR/Cas9 functional screens, several studies reported tumor cell intrinsic factors in immunotherapy resistance pathways in models of melanoma and leukemia [58, 93–95]. From these studies, chromatin structure modulators such as Ezh2 and PBAF complex components were revealed to be integral to the tumor cell/T cell interaction. Studies in multiple cancer types have validated the effect of Ezh2 on MHC class I antigen presentation [57, 58, 96], including HNSCC [56]. In contrast, the role of PBAF (Polybromo-associated BAF complex) in tumor cell interferon gamma sensitivity was only demonstrated in melanoma models [93]. Although further studies are needed, these findings suggest both cancer-type specific and conserved common epigenetic-related immune modulators exist, and defining their respective relative contribution will impact therapeutic target identification.

To specifically identify epigenetic modulators involved in the sensitivity or resistance of cancer immunotherapy, an epigenetic-focused *in vivo* screen was also performed using lung adenocarcinoma models [97]. Compared with *in vitro* screens, *in vivo* screens provide relatively more physiological conditions with endogenous antitumor immunity and immunotherapy treatment as selection pressure. In addition, in the complex tumor microenvironment, the interaction between cancer cells and immune cells is also not limited to “two-cell-type” coculture of the *in vitro* screening system. On the other hand, *in vivo* screens require large numbers of mice especially with unbiased screening libraries, which can be labor intensive and cost prohibitive compared to *in vitro* screens [95]. *In vivo* T cell CRISPR screens have also been shown to be feasible in immunotherapy target discovery [98]. Therefore, CRISPR/Cas9 screens using HNSCC models represent a valid strategy for identifying immune modulators involved in the resistance or sensitivity pathways of HNSCC to immunotherapy.

## Perspectives in HNSCC management

As discussed earlier, despite the durable response demonstrated with immune checkpoint inhibitors in patients with recurrent/metastatic HNSCC, various combinatorial treatment strategies, including with epigenetic targeting, are being actively tested to facilitate the antitumor immune response. Rodriguez and colleagues completed a Phase II trial of pembrolizumab and vorinostat in two distinct head and neck tumors-recurrent/ metastatic HNSCCs and salivary gland cancers [99]. For the HNSCC cohort, grade 3 AEs of any cause were observed in nine (36%) patients, which is higher than that reported for pembrolizumab alone. Although there were several limitations, including cohort size and heterogeneity and that there was no run-in phase of vorinostat alone, there was a 32% overall response rate that warrants further exploration. A second ongoing clinical trial is assessing whether addition of azacitidine to a durvalumab/tremelimumab combination will be safe and improve outcomes in recurrent/metastatic HNSCCs who have progressed on anti-PD-1, anti-PD-L1, or anti-CTLA-4 monotherapy (NCT03019003). There are several trials integrating EZH2 targeting with checkpoint inhibition (for example NCT03525795, NCT03854474) but these have not expanded to include HNSCCs. Our preclinical work represents a rational basis to complete such a study. Future studies may also integrate targeting of specific protein methyltransferases/demethylases that are associated with the immune-cold phenotype of HPV-negative HNSCC and could be considered for preclinical investigation to decipher mechanisms of CD8+ T cell exclusion in this disease **(**PMID: 29348866). Finally, patient selection with epigenetic biomarkers to define susceptible patients should be a goal of future clinical trials.

## Conclusion

In conclusion, recent preclinical studies have provided significant rationale in supporting the proposed treatment strategy of combining epigenetic targeting and immune checkpoint blockade in HNSCC to enhance treatment efficacy. The cell context-specific functions of epigenetic regulators and their impact on immunogenicity and synergy with immune-oncology approaches remain to be vigorously investigated preclinically in HNSCC. We have reviewed the impact of epigenetic targeting on various immune components involved in the tumor/host immune interaction. We believe that the epigenetic targeting combinatorial therapy with immune checkpoint blockade, especially *via* EZH2 inhibition, presents a robust opportunity for clinical HNSCC management. In parallel, novel epigenetic therapeutic target identification in preclinical HNSCCs represents an important frontier in advancing the field of HNSCC immunotherapy.
